# Development and Evaluation of Monoclonal Antibodies for Paxilline

**DOI:** 10.3390/toxins7103903

**Published:** 2015-09-25

**Authors:** Chris M. Maragos

**Affiliations:** Bacterial Foodborne Pathogens and Mycology Research Unit, USDA-ARS-NCAUR, 1815 North University Street, Peoria, IL 61604, USA; E-Mail: chris.maragos@ars.usda.gov; Tel.: +1-309-681-6266; Fax: +1-309-681-6672

**Keywords:** paxilline, tremorgen, antibody, immunoassay, mycotoxin, silage

## Abstract

Paxilline (PAX) is a tremorgenic mycotoxin that has been found in perennial ryegrass infected with *Acremonium lolii*. To facilitate screening for this toxin, four murine monoclonal antibodies (mAbs) were developed. In competitive indirect enzyme-linked immunosorbent assays (CI-ELISAs) the concentrations of PAX required to inhibit signal development by 50% (IC_50_s) ranged from 1.2 to 2.5 ng/mL. One mAb (2-9) was applied to the detection of PAX in maize silage. The assay was sensitive to the effects of solvents, with 5% acetonitrile or 20% methanol causing a two-fold or greater increase in IC_50_. For analysis of silage samples, extracts were cleaned up by adsorbing potential matrix interferences onto a solid phase extraction column. The non-retained extract was then diluted with buffer to reduce solvent content prior to assay. Using this method, the limit of detection for PAX in dried silage was 15 µg/kg and the limit of quantification was 90 µg/kg. Recovery from samples spiked over the range of 100 to 1000 µg/kg averaged 106% ± 18%. The assay was applied to 86 maize silage samples, with many having detectable, but none having quantifiable, levels of PAX. The results suggest the CI-ELISA can be applied as a sensitive technique for the screening of PAX in maize silage.

## 1. Introduction

**Figure 1:**
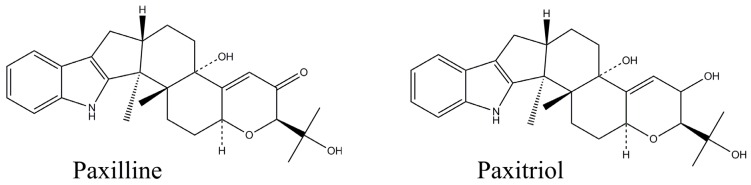
Structures of paxilline (MW 435.56) and paxitriol (MW 437.57).

The tremorgens are a class of fungal secondary metabolites (mycotoxins), which, like the ergot alkaloids, contain a substituted indole-moiety. There are many examples of tremorgens, including the penitrems (tremortins), janthitrems, lolitrems, aflatrem, paxilline, paspalines, paspalitrems, fumitremorgens, tryptoquivalines, verruculogen, and others [1]. They are produced by certain species of *Aspergillus*, *Penicillium*, *Claviceps,* and *Acremonium* [2,3,4,5]. Paxilline (PAX, Figure 1) was first isolated as a metabolite of *Penicillium paxilli* (*P. paxilli*) that caused sustained tremors and hypersensitivity to sound when given orally to one-day old cockerels and intra-peritoneally to mice [6]. PAX is a potent inhibitor of high conductance calcium-activated potassium channels [7,8], and has profound effects on the electromyographic activity of smooth muscle of the reticulorumen of sheep [9]. Lolitrem B, a related tremorgen, also inhibits such channels and this effect is believed to be the mechanism of action for the disease “ryegrass staggers” [10]. Paspaline, paspalicine, and paspalinine are structurally similar to PAX [11]. The group is variously known as the paspaline group [2], paspalitrem group [3], or the paspalanes [1]. Members of the group have been associated with Dallisgrass poisoning (aka “paspalum staggers”) in grazing cattle [11]. In addition to neurotoxicity, PAX has been shown to be genotoxic and to cause DNA damage in human lymphocytes [12,13].

PAX was isolated from *P. paxilli* obtained from insect-damaged pecans [6] and has been found in moldy tomatoes [14]. However, it is the occurrence of lolitrem B and PAX in perennial ryegrass that has been associated with disease, in particular “ryegrass staggers” in livestock [15]. PAX was found at significant levels (up to 14 mg/kg) in samples of ryegrass [16]. It is produced through the intermediate 3-geranylgeranylindole [17], and the biosynthesis has been recently summarized [18]. The chemistry of many of the tremorgens as well as their structural and biosynthetic relationships were well summarized by Cole [3] and Sings and Singh [1].

Perhaps because of the large number of possible tremorgens and the lack of widely available analytical standards for most of them, the development of analytical methods for this group of mycotoxins has not progressed to the same extent as for other common mycotoxins. Most of the early methods for detection of the paspalitrem-type mycotoxins were based upon liquid/liquid partitioning followed by thin layer chromatography (TLC), as summarized by Selala *et al.* [19]. PAX absorbs in the ultraviolet (UV) region and in methanol (MeOH) demonstrates absorption bands at 230 nm (ε 41,500) and 281 nm (ε 8000) [6]. For this reason, liquid chromatographic (LC) methods have incorporated UV or diode array detectors [14,19,20]. Upon exposure to UV light, PAX yields uncharacterized fluorescent products, with excitation maximum at 360 nm and emission maximum at 462 nm, which suggests LC with fluorescence detection is also possible [21]. Liquid chromatography with mass spectrometric detection (LC-MS) has been used to detect PAX in perennial ryegrass [22]. More recently, LC combined with high resolution MS has been applied to determine the idole-diterpenoid profiles of certain *Claviceps* species [23]. A screening assay for 186 fungal and bacterial metabolites in indoor matrices using LC with electrospray tandem ionization mass spectrometry (LC-MS/MS) also included PAX [24].

Antibodies for PAX were developed by AgResearch in New Zealand in the 1990s [16]. The antibodies were applied in enzyme-linked immunosorbent assays (ELISAs), and in combinations of TLC and LC with immunochemical detection [16,25,26]. Those appear to be the only published reports of such assays, although recently a commercial biosensor array has incorporated PAX, with a limit of detection (LOD) of 50 µg/kg [27]. Unfortunately, additional specifics of that assay have not been published. The objectives of our research were to develop antibodies and immunoassays for PAX and apply them towards a small-scale survey of PAX in maize silages.

## 2. Results and Discussion

### 2.1. Production of mAbs to PAX

**Figure 2:**
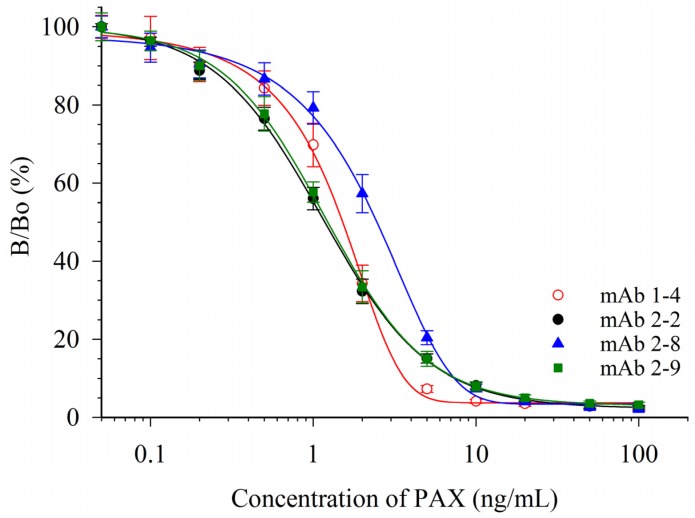
Response of four mAbs in CI-ELISA of PAX. Data shown are the averages of six plates ±1 standard deviation (SD).

Ten mice were immunized with a conjugate of paxitriol-hemiglutarate and ovalbumin (RPAX-OVA). Sera were evaluated with a competitive indirect ELISA (CI-ELISA). In this format an immobilized paxilline-bovine serum albumin (PAX-BSA) conjugate competed with free PAX for PAX antibodies. Two of the immunized mice were selected for splenocyte fusions and a total of 15 PAX-responsive cultures were obtained. From these, four antibody-producing monoclonal cell lines were isolated. These were designated mAb 1-4 (isotype IgG_1κ_), 2-2 (IgG_1λ_), 2-8 (IgG_1λ_), and 2-9 (IgG_2ακ_). Responses of these mAbs in competitive indirect enzyme-linked immunosorbent assays (CI-ELISAs) are depicted in Figure 2.

Calibration curves of PAX in phosphate buffered saline (PBS) were used to determine the concentrations needed to inhibit color development by 20% (IC_20_), 50% (IC_50_) and 80% (IC_80_) (Table 1). The response curves of mAbs 2-2 and 2-9 were essentially superimposable. Although the response curves were similar, the antibodies were distinctly different, as they had different isotypes (IgG_1λ_ and IgG_2ακ_). While not as sensitive to PAX, the shapes of the curves from mAbs 1-4 and 2-8 had steeper slopes. For quantitative assays, this resulted in a lower dynamic range for the assays with these two antibodies. A widely used measure of dynamic range for competitive immunoassays is the range of concentrations between the IC_20_ (minimum) and IC_80_ (maximum).

**Table 1:** Response parameters for four PAX mAbs in CI-ELISA (data from Figure 2).

Antibody	IC_20_ (ng/mL)	IC_50_ (ng/mL)	IC_80_ (ng/mL)	Dynamic Range (ng/mL)
1-4	0.6	1.5	2.7	0.6–2.7
2-2	0.4	1.2	3.6	0.4–3.6
2-8	0.9	2.5	5.2	0.9–5.2
2-9	0.4	1.2	3.6	0.4–3.6

Based upon the parameters in Table 1, mAb 2-9 was chosen as the antibody to use for further ELISA development. However, it should be noted that the attributes of mAb 1-4, with a similar IC_50_ but much steeper dose-response curve (and lower IC_80_), might make this a better choice of an antibody for a qualitative immunoassay format, such as for a lateral flow device. The sensitivity of these CI-ELISAs compares well to the previous literature. Garthwaite *et al.* [16] immunized mice with a PAX-*O*-(carboxymethyl)oxime-keyhole limpet hemocyanin conjugate and developed a CI-ELISA using immobilized PAX-ovalbumin. Several mAbs were isolated, and two were described in the publication. Although the IC_50_s were not provided, the dynamic ranges were reported. These were 2 to 1000 ng/mL (for mAb M-03/01) and 5 to 500 ng/mL (for mAb M-03/02) [16]. The dynamic ranges of the four mAbs reported herein were not as broad, however the assays were more sensitive (Table 1).

### 2.2. Effects of Solvents on mAb 2-9 ELISA

PAX is typically extracted using halogenated solvents or aqueous mixtures of organic solvents. Because of the toxicity of chlorinated solvents, the use of alternative organic solvents is preferable. Accordingly, the effects of acetonitrile (ACN) and MeOH on the mAb 2-9-based assay were determined. PAX standards were prepared in mixtures of PBS with solvent at different concentrations then assayed by CI-ELISA (Table 2).

**Figure 3:**
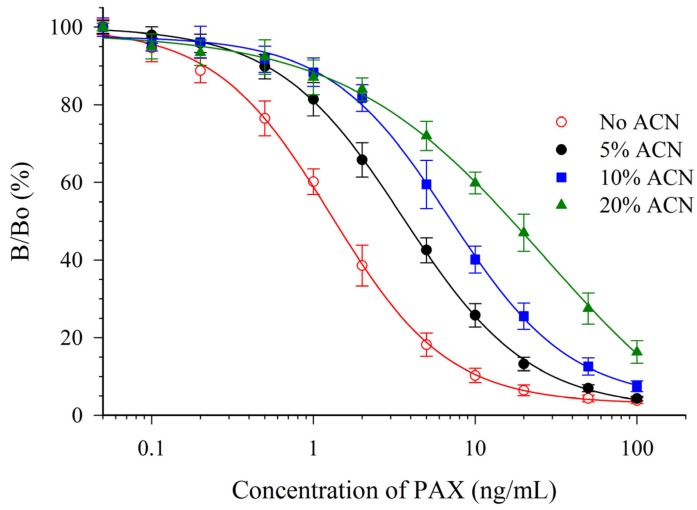
Effect of ACN upon the CI-ELISA with mAb 2-9.

**Figure 4:**
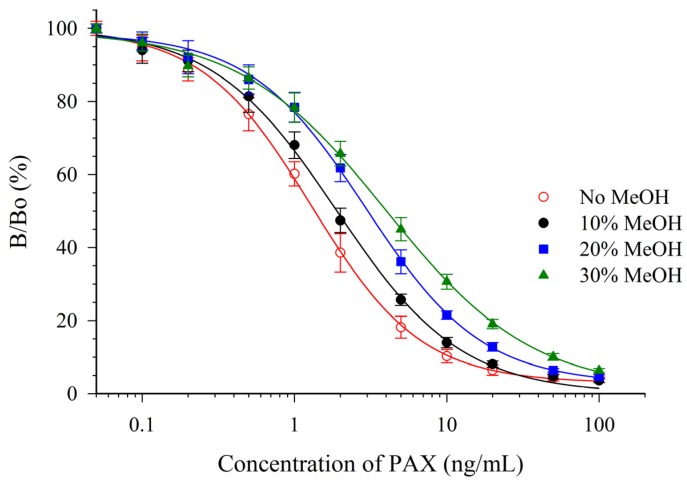
Effect of MeOH upon the CI-ELISA with mAb 2-9.

Both solvents were detrimental to the sensitivity of the assay. However, the antibody appeared to be much more susceptible to the effects of ACN (Figure 3) than to MeOH (Figure 4). One parameter for assessing the impact on the sensitivity is the relative effect of the solvent on the IC_50_ (e.g., relative response, Table 2). A relative response of 50% indicates that the IC_50_ has increased by two-fold (sensitivity has diminished two-fold). For ACN this occurred at a concentration below 5%, whereas for MeOH it occurred at a concentration between 10% and 20%. This suggested that MeOH, rather than ACN would be preferred as an extraction solvent. The mAb 2-9 ELISA appears to have tolerance to MeOH that is similar, or slightly less, than that reported previously for mAbs M-03/01 and M-03/02, which tolerated up to 15% [16]. The mAb 2-9 ELISA did not respond to the indole-containing toxins penitrem-A (PEN-A), or cyclopiazonic acid (CPA), or to the ergot alkaloid ergometrine, when tested at concentrations up to 10 µg/mL. The low cross-reactivities (<0.02%) demonstrated that the immunoassay was very selective for PAX.

**Table 2:** Effects of MeOH and ACN on the CI-ELISA.

PAX In:	Solvent Concentration ^a^	IC_50_ (ng/mL) ^b^	Relative Response (%) ^c^	*N* ^d^
PBS	0%	1.36 ± 0.16	100%	20
Methanol	10%	1.87 ± 0.21	72%	6
	20%	3.03 ± 0.28	44%	6
	30%	4.03 ± 0.52	33%	6
Acetonitrile	5%	3.72 ± 0.51	36%	6
	10%	7.2 ± 1.1	18%	6
	20%	17.3 ± 3.3	7%	6

^a^ Volume percentage of the solvent used to prepare the standards. Because the standards were mixed (1 + 1) with mAb in the assays, the concentration of solvent present during the competitive step was one half of the concentrations listed; ^b^ Average IC_50_ ± 1 SD for PAX; ^c^ Response of PAX in the indicated solution relative to PAX in PBS. See Experimental Section 3.5, Equation (1); ^d^ Number of ELISA plates used to determine the summary statistic.

### 2.3. Application of CI-ELISA to Silage Samples

**Figure 5:**
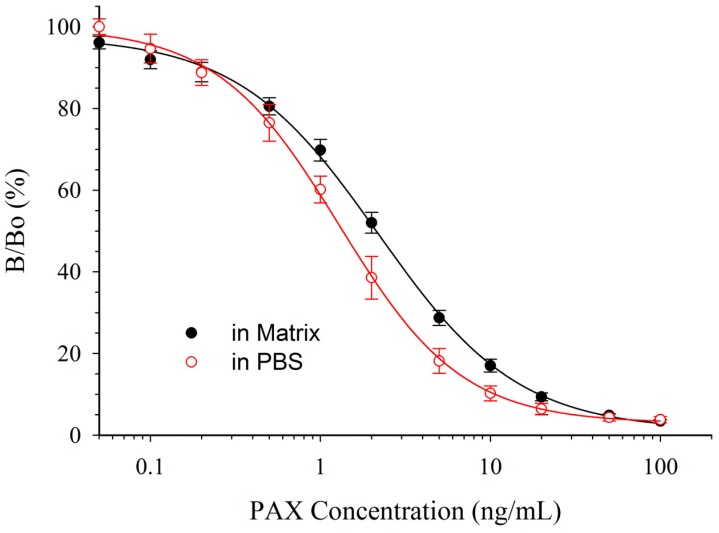
Effect of matrix, from ACN/H_2_O extracted silage, on the CI-ELISA with mAb 2-9. Data in matrix are the averages of 12 plates ±1 SD. Data in PBS are the averages of 20 plates ±1 SD.

The simplest way to remove solvent effects is by dilution, so in preliminary experiments ACN/H_2_O or MeOH/H_2_O extracts of silage received only dilution as sample pre-treatment prior to assay. However, matrix effects were too great to allow accurate quantification (data not shown). Therefore, the use of columns to cleanup the extracts before ELISA was evaluated. The columns, a mixture of silica bonded C-18, neutral aluminum oxide, Florisil, and celite removed many of the potential interferences while allowing the PAX to pass through un-retained. In HPLC experiments (data not shown), the cleanup columns worked well with ACN/H_2_O extracts of silage but poorly with MeOH/H_2_O extracts of silage. Therefore, despite the lower tolerance of mAb 2-9 to ACN, ACN/H_2_O was chosen as the extraction solvent. The responses of PAX calibration curves prepared in PBS and in diluted control matrix after SPE clean up are depicted in Figure 5.

Unfortunately, even with the sample cleanup, it was apparent that standards in PBS would not provide accurate quantification of silage extracts. In matrix, the IC_50_ was 2.13 ± 0.20 ng/mL, compared to 1.36 ng/mL in PBS (*i.e.*, a relative response of 64%). Much, if not all, of the effect was likely due to the ACN content of the extract. The percentage of ACN carried over into the test solution was 2.1% by volume. Since 5% ACN increased the IC_50_ to 3.72 ng/mL (*i.e.*, relative response of 36%, Table 2), it seems likely that the 2% ACN carried over into the diluted extract was still able to negatively affect the assay.

Because the objective of this work was to develop a rapid assay for PAX, and steps to remove all of the ACN would have been very time consuming, the use of matrix matched calibration curves (rather than solvent removal) was pursued. With matrix matched standards, the limit of detection (LOD), defined as the PAX concentration calculated to give a response three SD below the control signal, was 0.064 ng/mL. This was equivalent to 15 µg/kg dried silage. The limit of quantification (LOQ), calculated at 10 SD below the control signal, was 0.373 ng/mL, equivalent to 90 µg/kg in dried silage. Spiking and recovery experiments were conducted at five PAX levels, one between the LOD and LOQ and four above the LOQ (Table 3). The results indicated that the use of matrix matched standards allowed accurate quantification of PAX in dried silages over the range of 100 to 1000 µg/kg, with an average recovery over this range of 106% ± 18%.

**Table 3:** Recovery of PAX from spiked silage.

Spiking Level (µg/kg Dried Silage)	Recovery (% ±1 Std. Dev.) ^a^
100	106 ± 28
200	109 ± 18
500	106 ± 11
1000	102 ± 15

^a^ Percentage recovery ±1 SD (*n* = 4). Results from spiking at 50 µg/kg (below the estimated LOQ of 90 µg/kg) were 114% ± 26%.

The CI-ELISA was applied to 86 samples of maize silage that had previously been tested for fumonisin content [28]. While PAX was detected in many of the samples, in none of the samples was the average concentration found that exceeded the LOQ. Therefore, for the sample set tested, no significant levels of PAX were found.

## 3. Experimental Section

### 3.1. Reagents

Chicken egg albumin (OVA), polyvinyl alcohol (PVA), and ergometrine (also known as ergonovine) were purchased from Sigma-Aldrich (Milwaukee, WI, USA). Peroxidase conjugated goat anti-mouse IgG was purchased from Jackson ImmunoResearch Laboratories, Inc. (West Grove, PA, USA). PAX and penitrem-A (PEN-A) were obtained from Santa Cruz Biotechnology (Dallas, TX, USA). Cyclopiazonic acid (CPA) was manufactured by MP Biochemicals, LLC (Solon, OH, USA). Bonded C-18, 35–75 µm, 150 Å was obtained from Analtech (Newark, DE, USA). Neutral aluminum oxide, Brockman I, 50 to 200 µm, 60 Å, was obtained from Fisher Scientific (Pittsburgh, PA, USA), as was Florisil (60 to 100 mesh). Two-milliliter bed-capacity disposable polystyrene columns with frits (product number 29920), Celite 545, and bovine serum albumin (BSA) were also obtained from Fisher Scientific.

### 3.2. Ethical Statement

All animal procedures reported herein were approved by the Institutional Animal Care and Use Committee of Harlan Bioproducts for Science. Work was performed under protocol HAP 421-09—Rodent Immunization for Antibody Production (last review date 19 December 2014, next review in December 2015), in accordance with guidelines established by the National Institutes of Health-Office of Laboratory Animal Welfare.

### 3.3. Development of PAX mAbs

Because of the small size of PAX, a conjugate with OVA was prepared for immunization of mice. PAX was first reduced to paxitriol (Figure 1) with sodium borohydride [29]. The hemiglutarate of paxitriol (RPAX-HG) was prepared by adapting the method of Lau *et al.* [30] using glutaric anhydride and 4-*N*,*N*-dimethylaminopyridine. The products were extracted with ethylacetate and examined by mass spectrometry. The mass spectrometer (MS) used was an LQQ (Thermo Fisher Scientific, Waltham, MA, USA) equipped with an electrospray ionization source and operated in negative ionization mode (−ESI). Samples were sprayed using a 4.20 kV spray voltage, and optimized parameters of the system for MS detection were: inlet capillary voltage: −55 V; tube lens voltage: −10 V; capillary temperature: 215 °C. All analyses were conducted with a mass scan range of 150–2000 Da. A portion of the ethylacetate extract was diluted in MeOH and infused into the ESI source with a syringe pump at a flow rate of 18 μL/min. The ethylacetate extract was found to contain a mixture of paxitriol (*m*/*z* 436.3), RPAX-HG (*m*/*z* 550.1) and a dimer of RPAX-HG (*m*/*z* 1101.3). This mixture was used to prepare a RPAX-HG-OVA conjugate (RPAX-OVA) using the well established mixed anhydride reaction [30]. To prepare a solid-phase antigen for use in screening assays, PAX was also conjugated to BSA. The *O*-carboxymethyloxime of PAX (PAX-CMO) was first prepared by the method of Chu *et al.* [31] using carboxymethoxylamine hemihydrochloride. The products were extracted with ethylacetate and examined by −ESI. The organic phase was a mixture of PAX-CMO (*m*/*z* 507.1) and a dimer of PAX-CMO (*m*/*z* 1015.1). This mixture was used to prepare a PAX-CMO-BSA conjugate (PAX-BSA), also using the mixed anhydride reaction [31]. The RPAX-OVA and PAX-BSA were dialyzed extensively to remove unbound paxitriol or PAX-CMO. Conjugates were diluted to 2 mg/mL with 0.1 M PBS, then freeze-dried and sent to Harlan Bioproducts for Science (HBPS, Madison, WI, USA) for immunization of mice and collection of sera. Ten female Balb/C mice were initially immunized by injection of 100 µg RPAX-OVA per animal using the same procedures as described previously for production of T_2_-glucoside antibodies [32]. A CI-ELISA was developed and used for screening of mouse sera and culture supernatants for the presence of antibodies. Each hybridoma culture supernant solution was tested in single replicates at a minimum of three dilutions, and positive cultures were re-assayed. For screening assays, 0.1 mL of PAX-BSA (1 μg/mL in 0.05 M sodium phosphate buffer, pH 7), was added to wells of polystyrene microtiter plates and allowed to attach overnight at 4 °C. After washing the coated plate twice with 0.32 mL Tween-PBS (0.02% *v*/*v* Tween-20 in 0.01 M PBS pH 7), 0.32 mL of PVA-PBS (1% *w*/*v* PVA in 0.01 M PBS) was added and allowed to incubate at ambient temperature for 2 h. During this incubation, test solutions were prepared. The test solutions consisted of 0.1 mL of toxin standard solution (or PBS control) mixed with 0.1 mL of serum (or culture fluid) diluted in BSA-PBS (1% *w*/*v* BSA in 0.01 M PBS) in the wells of a polypropylene microwell plate (Corning Inc., Corning, NY, USA). The wells of the polystyrene (PAX-BSA immobilized) plate were washed twice with Tween-PBS and 0.1 mL of test solution was transferred into each well. After incubation at ambient temperature for 30 min the plate was washed three times and 0.1 mL of goat anti-mouse peroxidase conjugate (diluted 1:2000 in BSA-PBS) was added. The plate was incubated for 30 min at ambient temperature then washed four times before addition of 0.1 mL of the *O*-phenylenediamine (OPD) substrate. The OPD solution was prepared by combining 0.02 mL of 30% H_2_O_2_ and 20 mg OPD in 50 mL of citrate-phosphate buffer (0.05 M citrate, 0.1 M phosphate, pH 5.0). After 5 min at ambient temperature, the reaction was stopped by the addition of 0.1 mL of 1 N hydrochloric acid. Color development was determined by measuring the absorbance at 490 nm using a Synergy HT microplate reader (Bio-Tek, Winooski, VT, USA).

Two mice having sera with antibodies reactive to PAX were sacrificed and aseptically splenectomized at Harlan Bioproducts for Science (Madison, WI, USA). Spleenocytes were chemically fused with Balb/C non-immunoglobulin secreting (NS-1) myeloma cells using polyethylene glycol. Fused cells were plated in HAT selection media. After 11 days, HAT resistant cultures were isolated and screened for anti-PAX activity by CI-ELISA. The two fusions yielded a total of 15 positive cultures. From these four cultures were subsequently cloned, expanded and frozen. The cultures were designated 1-4.1.16 (herein “1-4”), 2-2.1.2.2.1.1.1.10 (herein “2-2”), 2-8.2.2.1.2 (herein “2-8”), and 2-9.1.8.2 (herein “2-9”). With this nomenclature, the first digit describes the fusion and the second digit describes the hybridoma cell line derived from that fusion. These four cell lines were expanded to produce ascites fluid in mice using established procedures [33]. The ascites fluid was partially purified by ammonium sulfate precipitation using procedures described previously [34], then lyophilized. Protein content of each of the preparations was determined using the BCA Protein Assay according to the protocols provided by the manufacturer (Thermo Fisher, Pittsburgh, PA, USA).

### 3.4. Comparison of the PAX mAbs by CI-ELISA

Preliminary experiments were used to establish the PAX concentration range needed to determine the IC_50_s. The CI-ELISA protocol was the same as that described for screening of the cultures, with the exception that less PAX-BSA was immobilized (0.1 µg/mL). A stock solution of PAX was prepared gravimetrically at a concentration of 1 mg/mL in ACN. The concentration was verified by diluting an aliquot in MeOH, with reference to the published extinction coefficient of 41,500 at 230 nm [6]. For calibration curves, the PAX standards were prepared by serial dilution of the stock solution with ACN (for concentrations of 10 µg/mL and higher), ACN/PBS (1 + 9 *v*/*v*, for the 1 µg/mL intermediate dilution), or PBS (for concentrations less than 1 µg/mL). Calibration curves covered the range from 0.1 to 100 ng/mL (0.23 to 230 nM). Because equal volumes of standard and mAb were mixed in these assays, the actual concentrations present in the competition mixture were one-half of these values (*i.e.*, 0.05 to 50 ng/mL). To compare the four mAbs, the antibody preparations were diluted so that, in the absence of PAX, the absorbance developed (*i.e.*, B_o_) was in the range of 1.1 to 1.4. This corresponded to dilutions of 1:5000 (1-4 and 2-8) or 1:10,000 (2-2, 2-9). Comparisons were made on 6 replicate plates, with duplicate wells per antibody/toxin combination per plate.

### 3.5. Evaluation of Solvent Tolerance

To evaluate the impact of solvent upon the CI-ELISA with mAb 2-9, assays were conducted with PAX standards prepared in either 0.01 M PBS (e.g., “solvent free”), mixtures of MeOH and PBS (10% to 30% by volume) or mixtures of ACN and PBS (5% to 20% by volume). The PBS and solvent/PBS combinations were tested together (side-by-side) on the same plates. Six replicate plates were assayed for the MeOH/PBS combinations and 6 for the ACN/PBS combinations. An additional 8 plates were assayed with standards in PBS alone (from the mAb comparisons, see above), therefore data for PBS were from a total of 20 plates. Data were transformed by dividing the measured absorbance (B) by the absorbance of toxin-free control (B_o_) and represented as a percentage. The transformed data were fit using a 4-parameter logistic dose-response equation (TableCurve, Systat Software, Inc., San Jose, CA, USA). Fitted curves were used to determine the individual IC_50_s. The IC_50_s were used to estimate the response of standards in solvent relative to standards in PBS.

Relative Response in Solvent = (IC_50_ in PBS)/(IC_50_ in solvent solution) × 100% (1)

The cross-reactivity of the mAb 2-9 to CPA, PEN-A, and ergometrine were also evaluated. Stock solutions were prepared gravimetrically at 1 mg/mL in acetonitrile. Intermediate dilutions at 0.1 mg/mL were prepared in ACN/PBS (1 + 3, *v*/*v*). Serial dilutions were prepared in PBS over the range from 10 to 10,000 ng/mL and tested alongside PAX in CI-ELISA.

### 3.6. Application to Silages

Samples of maize silage were collected from the Midwestern United States during 2001–2002 and were provided by a seed company as part of a previous study on fumonisins in silage [28]. None of the silages were associated with health problems or were considered to be of unusually poor quality. The sample size was circa 1–2 kg. The samples were divided into three sections. Both end sections (*ca.* 10 cm length), which were exposed to air and were visibly molded for some samples, were removed. All silage samples were dried for 48 h at 45–55 °C in a convection oven. The dried samples were ground to a fine consistency in a Stein Laboratory Mill (The Stein Corp., Atchison, KS, USA) and stored at −20 °C prior to analysis.

A sorbent mixture of silica bonded C-18, neutral aluminum oxide, Florisil, and Celite 545 was prepared in the following proportion (by weight): 9:120:80:16. This was thoroughly mixed and 1.25 g of sorbent was poured into 2 mL (5 mm diameter) disposable columns containing a frit, then pressed into place with a top frit. Five grams of dried silage was extracted with 30 mL of ACN/H_2_O (84 + 16 *v*/*v*) by shaking for 1 h on a wrist action shaker at ambient temperature. The extraction mixture was briefly allowed to settle and 3.5 mL of liquid was transferred to the top of the cleanup column. The first 2 mL of eluate was collected from the column for analysis. For analysis by CI-ELISA, a 125-µL aliquot of the purified extract was diluted 1:40 (*v*/*v*) with 0.01 M PBS. For recovery studies, PAX solution (10 µg/mL in ACN) was added to 5 g of dried silage and stored overnight at ambient temperature to allow the solvent to evaporate, then extracted as described above. The 5 spiking levels tested and the volumes of stock solution used for the spiking were: 1000 µg/kg (500 µL), 500 µg/kg (250 µL), 200 µg/kg (100 µL), 100 µg/kg (50 µL), and 50 µg/kg (25 µL).

To evaluate the impact of matrix upon the CI-ELISA with mAb 2-9, assays were conducted with PAX standards prepared in either 0.01 M PBS or the purified extract from a control silage sample (containing no detectable PAX). For the recovery studies, the concentration in spiked samples was determined from a matrix matched calibration curve constructed over the concentration range of 0.05 to 100 ng/mL. Four replicate plates were assayed, with quadruplicate wells for each toxin level on each plate. The LOD was determined from 12 replicate plates by using the calibration curve to estimate the PAX concentration required to give a signal (B) three SD below the signal from the control (B_o_). The limit of quantification (LOQ) was estimated likewise, with a signal 10 SD below the control.

## 4. Conclusions

Four murine mAbs were developed for PAX, with IC_50_s in CI-ELISA that ranged from 1.2 to 2.5 ng/mL. While sensitive, the mAb 2-9 was also readily influenced by ACN or MeOH, with only 5% ACN or 20% MeOH resulting in a two-fold or greater increase in IC_50_. Aqueous ACN extracts of silage samples were cleaned up by adsorbing potential matrix interferences onto a solid phase column containing a sorbent mixture of silica bonded C-18, neutral aluminum oxide, Florisil, and Celite 545 and then diluted. Using this method, recovery from spiked silages was good, with an LOD of 15 µg/kg and an LOQ of 90 µg/kg. Recovery from dried silages spiked over the range of 100 to 1000 µg/kg averaged 106% ± 18%, indicating accurate quantification of PAX over this range. The assay was applied to 86 maize silage samples. Many had detectable, but none had quantifiable, levels of PAX, suggesting PAX levels in these otherwise normal silage samples were low.
